# Supplementary material for: Construction of a Necroptosis-Related lncRNA Signature for Predicting Prognosis and Immune Response in Kidney Renal Clear Cell Carcinoma
Source: Cells. 2022 Dec 23;12(1):66. doi: 10.3390/cells12010066 (PMC9818734; doi:10.3390/cells12010066)
Supplement: Supplementary file 1 [file cells-12-00066-s001.zip › SUPPLEMENTARY MATERIAL LEGENDS.pdf]

SUPPLEMENTARY MATERIAL

Figure S1. Heatmap of necroptosis-related DEGs between the normal and KIRC.

Figure S2. Forest plot showing prognostic necroptosis-related lncRNAs selected by univariate Cox regression analysis.

File S1. 258 necroptosis-related genes in the transcriptome data.

File S2. 709 necroptosis-related lncRNAs obtained from the TCGA.
