# Supplementary figures and images for: Construction of a Necroptosis-Related lncRNA Signature for Predicting Prognosis and Immune Response in Kidney Renal Clear Cell Carcinoma
Source: Cells. 2022 Dec 23;12(1):66. doi: 10.3390/cells12010066 (PMC9818734; doi:10.3390/cells12010066)

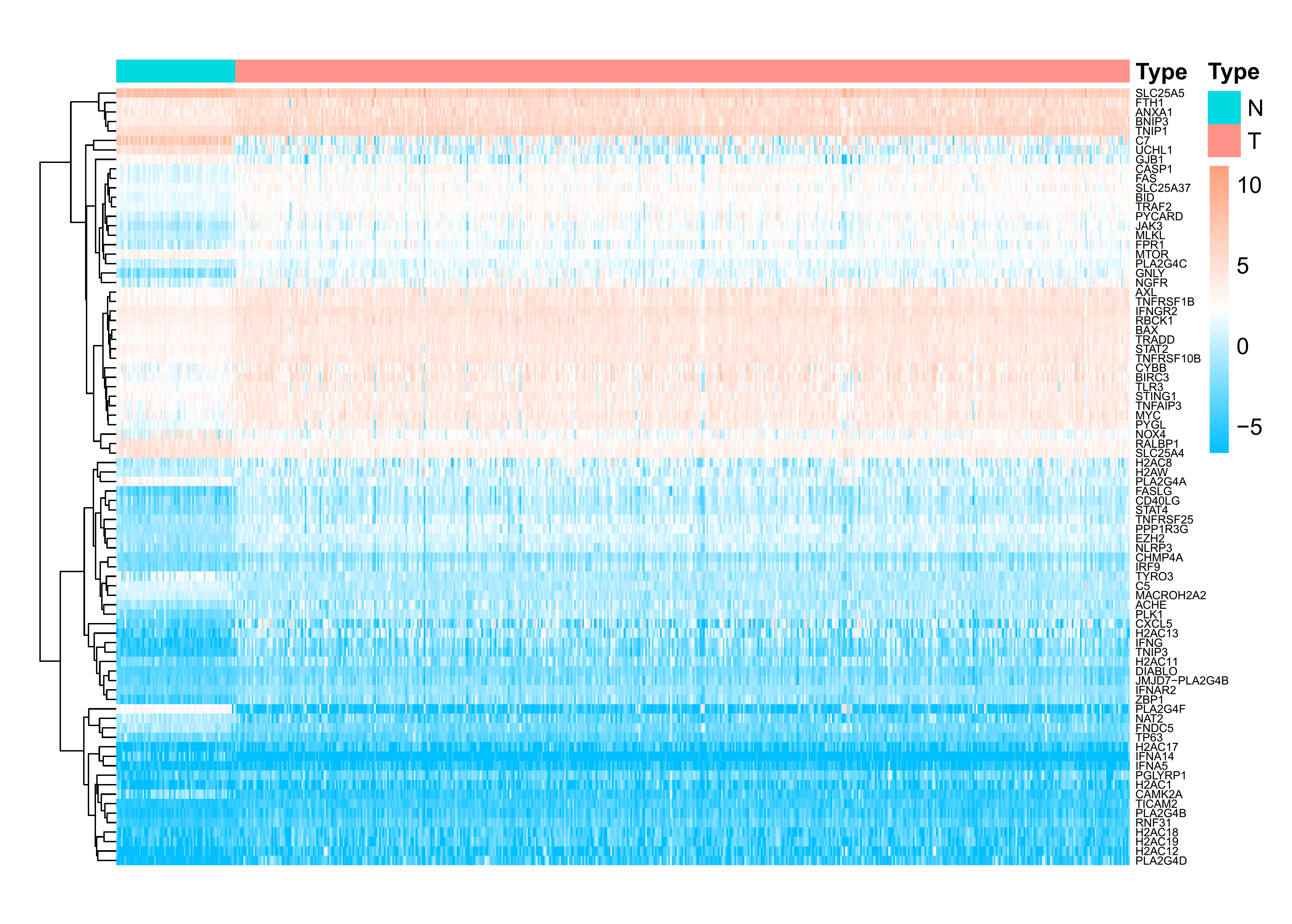

Supplement: Supplementary file 1 [file cells-12-00066-s001.zip › Figure S1.jpg]
